# Editorial: Strategies of Lymph Node Dissection During Sublobar Resection for Early Stage Lung Cancer

**DOI:** 10.3389/fsurg.2022.895806

**Published:** 2022-04-26

**Authors:** Monica Casiraghi, Lorenzo Spaggiari

**Affiliations:** ^1^Department of Thoracic Surgery, IEO, European Institute of Oncology IRCCS, Milan, Italy; ^2^Department of Oncology and Hemato-Oncology, University of Milan, Milan, Italy

**Keywords:** NSCLC, sublobar lung resection, lymphadenectomy, segmentectomy, lymph node

Non-small-cell lung cancer (NSCLC) is the major cause of cancer death, with a poor 5-year survival rate due to the small percentage of patients diagnosed with early-stage disease, and thus potentially being operable and curable (1). Thanks to diffusion of lung cancer screening programs, which are able to reduce lung cancer mortality up to 30% in high-risk subjects, in the last decades surgeons are pushed to deal with a greater number of small nodules and therefore with the need for new and different surgical strategies. Lately, thoracic surgery has been revolutionized by the development of minimally invasive techniques, such as video-assisted thoracoscopic surgery (VATS) and robotic-assisted thoracic surgery (RATS), but also by the increasing use of limited resection such as sublobar resection (SLR), which is gradually become the treatment of choice for early-stage NSCLC, instead of the traditional lobectomy performed via thoracotomy.

So far, the standard surgical treatment of NSCLC, even in cases of small nodules, has been lobectomy combined with lymph node (LN) dissection (LND) (2). However, looking at the literature it is clear that there is growing interest in SLR, and not only in patients who cannot tolerate a major resection (3), even if the results of these studies are still very controversial.

Despite increasing interest for SLR, different publications (4, 5) still show poor results on local recurrence and survival, regardless of the tumor size, compared to lobectomy, which instead provides satisfactory safety margins and allows removal of the lymphatic networks and intralobar nodes. Gossot et al. in their study showed that the possible reasons for the SLR oncological inferiority compared to lobectomy could be caused by different reasons: (1) some SLRs were wedge resections and not anatomical resections, (2) insufficient resection margins, (3) low number of nodes resected, (4) missing analysis of the so-called “adjacent” nodes, and (5) no frozen section on margins and on the segmental nodes.

On the other hand, many recent studies have demonstrated the non-inferiority of SLR over lobectomies for early-stage NSCLC (6–10), and nowadays, teams are increasingly performing SLR for selected cases of early-stage tumors.

However, as Gossot et al. stated in their study, it is very important, in case of SLR, to follow the same oncological rules for lobectomies, such as performing macroscopically complete resection with free margins and systematic LND, according to the guidelines (11). Insufficient LND during SLR that impacts oncological survival increases local recurrence and misjudging of the stage of the patients, resulting in an incorrect therapeutic approach and a worse survival. Different studies showed that survival was significantly better after lobectomy, except in the subgroup of SLRs associated with lymph node dissection, confirming the importance of a systematic hilar and mediastinal LND during segmentectomies (12–14).

In conclusion, SLR for early-stage NSCLC could be a valid alternative to lobectomy, but the same rules as for lobar resection, such as radical resection with free margins and adequate LND, must be applied.

## Author Contributions

MC designed and wrote the editorial. LS supervised the study. Both authors contributed to the article and approved the submitted version.

## Conflict of Interest

The authors declare that the research was conducted in the absence of any commercial or financial relationships that could be construed as a potential conflict of interest.

## Publisher's Note

All claims expressed in this article are solely those of the authors and do not necessarily represent those of their affiliated organizations, or those of the publisher, the editors and the reviewers. Any product that may be evaluated in this article, or claim that may be made by its manufacturer, is not guaranteed or endorsed by the publisher.
